# Public Health Note

**Published:** 1898-08

**Authors:** 


### PUBLIC HEALTH NOTE.

TYPHOID FEVER FROM ORDINARY STREAMS.—Recent reports to the Michigan State Board of Health show that at least two cases of typhoid fever have been caused by drinking the water in or from the Kalamazoo river. In no case should water be used for drinking or culinary purposes from a river or stream into which sewage is emptied or privies are liable to drain; and as most of the rivers and streams of this state have towns situated upon or near their banks, from which sewage is received and privies are liable to drain, therefore it is very dangerous to use the water from any of the rivers or streams for any culinary purpose whatever, except the water be first boiled, and it should not, in any case whatever, be drank unless previously boiled. Cases have been reported where typhoid fever has been contracted by bathing in streams. Probably this will be most likely to occur through accidentally or carelessly taking the infected water into the mouth. Especially should no person bathe in any ordinary stream just below any city, village or other source of sewage or privy drainage.

